# Chondroprotective Activity of *Murraya exotica* through Inhibiting ****β****-Catenin Signaling Pathway

**DOI:** 10.1155/2013/752150

**Published:** 2013-12-19

**Authors:** Longhuo Wu, Haiqing Liu, Rui Zhang, Linfu Li, Jialin Li, Haibo Hu, Hao Huang

**Affiliations:** College of Pharmacy, Gannan Medical University, Ganzhou 341000, China

## Abstract

Osteoarthritis (OA) is a degenerative joint disease that affects millions of people. Currently, there is no effective drug treatment for it. The purpose of this study is to investigate the chondroprotective effects of *Murraya exotica* (L.) on OA. The rat OA models were duplicated to prepare for separating OA chondrocytes, synovial fluid (SF), and serum containing *M. exotica* (50 mg/kg, 100 mg/kg, and 200 mg/kg), *M. exotica* showed the activity of decreasing the contents of TNF-**α** and IL-1**β** in SF and the chondrocyte apoptosis in a dose-dependent manner. To investigate the probable mechanism, quantitative real-time polymerase chain reaction (qRT-PCR) and western blotting were used to determine gene expression and protein profiles, respectively. The results reveal that *M. exotica* can downregulate mRNA and protein expressions of **β**-catenin and COX-2 and reporter activity significantly. Conclusively, *M. exotica* exhibits antiapoptotic chondroprotective activity probably through inhibiting **β**-catenin signaling.

## 1. Introduction

Osteoarthritis (OA) is a progressive joint disorder, which remains the leading cause of chronic disability in aged people. It had been elucidated that the signaling pathways directing joint formation and homeostasis were the key molecular players in OA [1]. Wnt proteins play central roles in a variety of developmental processes and events, including organogenesis, cell differentiation, morphogenesis, and tissue remodeling [2]. In the canonical Wnt/*β*-catenin signaling pathway, Wnt protein binds to cell-surface frizzled and the coreceptor low density lipoprotein receptor-related protein 5 and 6 (LRP-5/6), leading to inhibition of *β*-catenin phosphorylation by glycogen synthase kinase 3 beta (GSK-3*β*) and proteasome-mediated degradation; stabilized *β*-catenin translocates into the nucleus, where it interacts with resident lymphoid enhancer factor/T-cell (LEF/TCF) transcription factors to activate target genes [3].

Cumulating studies mainly based on experimental animal models for OA have suggested an important procatabolic role for Wnt/*β*-catenin signaling in the pathogenesis of OA [2, 4]. Direct genetic evidence for *β*-catenin in OA had not been reported, because tissue-specific activation of the **β*-catenin* gene (target by *Col2a1-Cre*) was embryonic lethal. In *Col2a1-CreER* ^*T2*^ *β*-*catenin* ^*fx*(*Ex*3)/*wt*^ mice, overexpression of *β*-catenin protein was detected by immunostaining in the 3th month, reduction of Safranin O and Alcian blue staining in the 5th month, and cell cloning, surface fibrillation, vertical clefting, and osteophyte formation were observed in the 8th month. In addition, expression of chondrocyte marker genes, such as *aggrecan*, *MMP-9*, *MMP-13*, *Alp*, *Oc*, *colX*, and *Bmp2,* was significantly increased [4]. *ColX*-expressing chondrocytes were detected in *Col2a1-Smurf2* transgenic mice which might represent a mechanism of Smurf2-induced OA that Smurf2 mainly induced ubiquitination of GSK-3*β* and its proteasomal degradation, and hence upregulation of *β*-catenin [5].

*Murraya exotica *(L.) (Rutaceae) is widely grown in the southern China, and it has been well documented in Pharmacopoeia of the People's Republic of China, 2010 Edition (Ch.P 2010) for treating stomachache, rheumatic arthralgia, toothache, body swelling, and pain [6]. In our previous studies, the 70% ethanol extracts of *M. exotica* show antinociceptive and anti-inflammatory activities in rat knee osteoarthritis models. It can downregulate the expressions of inducible nitric oxide synthase (iNOS), interleukin-1*β* (IL-1*β*), and tumor necrosis factor-*α* (TNF-*α*) in the rat serum significantly [7]. In this paper, we further investigated the changes of cytokines in the synovial fluid (SF) and explored culturing the OA chondrocytes in the separated rat serum containing *M. exotica*, which might influence the apoptosis and the *β*-catenin signaling in OA chondrocytes.

## 2. Materials and Methods

### 2.1. Plant Material

The *M. exotica* leaves employed in this study were collected at Zhangzhou (Fujian, China) in 2011. The plants were identified by Jialin Li. The voucher specimen (ID: GMU-M20081008) was deposited in the herbarium of College of Pharmacy, Gannan Medical University, Jiangxi Province, China. The leaves were harvested, air-dried, and then grounded into fine powder (150–200 mesh) with a laboratory scale mill.

### 2.2. General

Approximately 1 kg of the above-mentioned fine powder was extracted with 10 L of 70% ethanol for 48 h by maceration at room temperature. The extract was evaporated in vacuum to generate a crude ethanol extract (18.41%, w/w) [7]. The 70% ethanol extracts were dissolved in 0.8% sodium CMC in 50 mg/kg, 100 mg/kg, and 200 mg/kg doses, respectively. (100 mg/kg is the regular dose according to Ch.P 2010).

The study was approved by the Institutional Animal Care and Use Committee of Gannan Medical University. Each rat was intragastrically administered with the 70% ethanol extracts at different doses. The control group animals received the same experimental handling as those of the treating groups except that the drug treatment was replaced by appropriate volumes of the dosing vehicle. Indomethacin (10 mg/kg) was used as positive reference.

Preparation of rat serum containing *M. exotica* was as follows. Rats were intragastrically administered with the 70% ethanol extracts at 50 mg/kg, 100 mg/kg, and 200 mg/kg doses for one week, respectively. Rats were sacrificed, 5 mL of blood was taken from the heart, and serum was separated by centrifuge and ready for cell culture.

Before administration of *M. exotica*, cells were starved for 24 hours with serum-free medium. The separated rat serum containing *M. exotica* was added into cells and incubated for another 24 hours.

### 2.3. Rat Knee OA Model

Rat OA model was established by using Hulth's (1999) method [8]. The procedure is listed as follows. The rat was anesthetized with intravenous injection of 3% pentobarbitone (30 mg/kg). After a routine disinfection, 1 cm longitudinal incision was made at the medial parapatellar separating and cutting off the tibial collateral ligament, the articular cavity was opened and the cruciate ligament of knee was cut off, the medial meniscus was excised and the articular cavity was rinsed and sutured layer by layer, and then the rats underwent penicillin treatment for one week for prevention against infection. After 8 weeks since establishment of the model, the rats were sacrificed and the knee SF lavages were collected and kept at −20°C for ELISA determination of IL-1*β* and TNF-*α*. Other segments of the cartilage were taken for chondrocytes separation and culture.

### 2.4. Primary Cell Culture

Eight-week-old OA model group rats were sacrificed. Immediately, cartilage was harvested from the knee joint under sterile conditions as digested with 0.25% pancreatic enzymes for 30 min to remove other tissues and cells and then digested with 0.2% collagenase II at 37°C for 4 h. Cells were grown to confluence in DMEM (low glucose) supplemented with 10% fetal bovine serum (FBS) or rat serum containing *M. exotica*, 100 U/mL penicillin, and 100 mg/mL streptomycin at 37°C with 5% CO_2_. The chondrocytes were identified by toluene blue stain and type II collagen immunohistochemistry reaction. Cells from the first passage were used.

### 2.5. Quantitative Analysis of Apoptotic Cells

The changes of cell apoptosis were quantified by loading FITC annexin V/PI double—fluorescence labeling and using flow cytometry. Flow cytometry was performed according to the apoptosis detection kit (Nanjing KeyGEN Biological Technology Development Co., Ltd., Nanjing, China) procedures. After being treated by *M. exotica*, cells (1 × 10^6^/mL) were collected by centrifugation and incubated in buffer containing FITC annexin V and PI. Apoptotic cells were measured by a flow cytometer (FACSCalibur BD, San Jose, CA).

### 2.6. MTT Assays

OA chondrocytes were cultured in a 96-well plate (1 × 10^5^/mL). After incubation for 24 h in media containing different doses of *M. exotica*, MTT (5 mg/mL) was added (20 *μ*L/well). Cells were then incubated with MTT for 4 h, and DMSO (150 mL/well) was added after removing the culture medium. Absorbance was measured at 570 nm. This step was repeated for four times to get average results.

### 2.7. Gene Expression Analysis

Total RNA was extracted from chondrocytes using the Easy-spin total RNA extraction kit (iNtRON Biotechnology, Seoul, Korea). For each sample, 2 *μ*g of total RNA was reverse-transcribed using M-MLV (Promega, USA) to synthesize the first-strand of cDNA following standard protocols. To detect the expression level of COX-2, *β*-catenin, and caspase-3 genes, EzOmics SYBR qPCR kits were purchased from Biomics in a Mastercycler (Eppendorf). Their respective primer sequences (listed in Table 1) were used. Amplification procedure was as follows: 94°C for 5 min, followed by 30 cycles at 94°C for 30 s, 56°C for 45 s, 72°C for 45 s, and finally at 72°C for 10 min. The PCR reactions were performed using the Ani-Cycler real-time PCR system (Bio-Rad).

All of the PCR reactions were performed in sets of four. GAPDH was used as an internal control. Primer and template designs followed the same criteria for each target, and primers and Mg^2+^ concentrations had been optimized to render efficiency for each target near one per assumption underlying the 2^−ΔΔCT^ method [9].

### 2.8. Luciferase Reporter Assay

Chondrocytes were resuspended in serum-free culture medium and plated on 48-well dishes (3.4 × 10^4^ cells in 200 *μ*L/well) and transfected with Wnt/*β*-catenin reporter plasmid (Upstate, Lake Placid, NY) (Topflash, encoding seven copies of LEF/TCF binding sites linked to firefly luciferase and reflecting Wnt/*β*-catenin signaling activity) in the presence of Lipofectamine 2000. In all experiments, cells were cotransfected with Renilla luciferase plasmid (pRL-CMV; Thermo Fisher Scientific) to control for transfection efficiency. Cultures were transfected for 4 h, prior to addition of 200 *μ*L FBS containing media, and incubated overnight. On the next day, cells were administrated by *M. exotica* at different doses for 24 h. Cultures were then lysed with 1 × Passive Lysis Buffer (Promega, Madison, WI). The luciferase activities of both Topflash and pRL-TK-luc reporters were measured using a dual luciferase assay kit (Promega, Madison, WI) in an L-max II microplate reader (Molecular Devices, Sunnyvale, CA, USA).

### 2.9. Western Blot Analysis

Cells were lysed in lysis buffer (2% SDS, 10% glycerol, 10 mmol/L Tris, pH6.8, 100 mmol/L DTT) and then subjected to immunoblot. Before sampling, the protein concentrations were measured using a BCA Protein Assay Kit (Pierce Biotechnology, Rockford, IL, USA) with bovine serum albumin as a standard. After being combined with gel loading buffer (50 mmol/L Tris-HCl, pH6.8, 2% SDS, 10% glycerol, and 0.1% bromphenol blue) and boiled for 5 min, samples (80 *μ*g) were electrophoresed on 10% SDS-PAGE gel for anticleaved caspase 3, *β*-catenin, and COX-2. Proteins were western-blotted onto polyvinylidene difluoride (PVDF) transfer membranes, and blots were blocked with Tris-buffered saline (TBS) containing 5% nonfat milk for 1 h and incubated with anti-cleaved caspase 3, *β*-catenin, and COX-2 at 4°C overnight. The blots were then rinsed and incubated with HRP-conjugated IgG goat anti-rat for 1 h. The blots were then washed and developed by use of a Super Enhanced chemiluminescence detection kit (Applygen Technologies Inc., Beijing, China), and the protein bands were visualized after the exposure of the membranes to Kodak film (USA). GAPDH was used as the internal control in all Western blot analyses.

### 2.10. Statistical Analysis

All data were expressed as mean ± standard deviation (SD). Statistical analysis of gene expression data was analyzed by a paired *t*-test. Differences were considered significant at *P* < 0.05.

## 3. Results

### 3.1. *M. exotica* Decreased the Contents of Cytokines in SF

No statistically significant differences were observed in urea-adjusted synovial lavage concentration of IL-1*β* and TNF-*α* at the time of harvest (Table 2). However, the contents of IL-1*β* and TNF-*α* in the rats SF were decreased greatly in *M. exotica* group. At the dose of 100 mg/kg, the contents of IL-1*β* and TNF-*α* were 53.3 ± 10.8 pg/mL and 50.5 ± 11.4 pg/mL, respectively, which were slightly more effective than those in indomethacin group. In contrast, the model group showed the contents of IL-1*β* and TNF-*α* as 89.2 ± 14.8 pg/mL and 80.3 ± 11.6 pg/mL, respectively.

### 3.2. Cell Culture and Apoptotic Analysis

**Figure 1:**
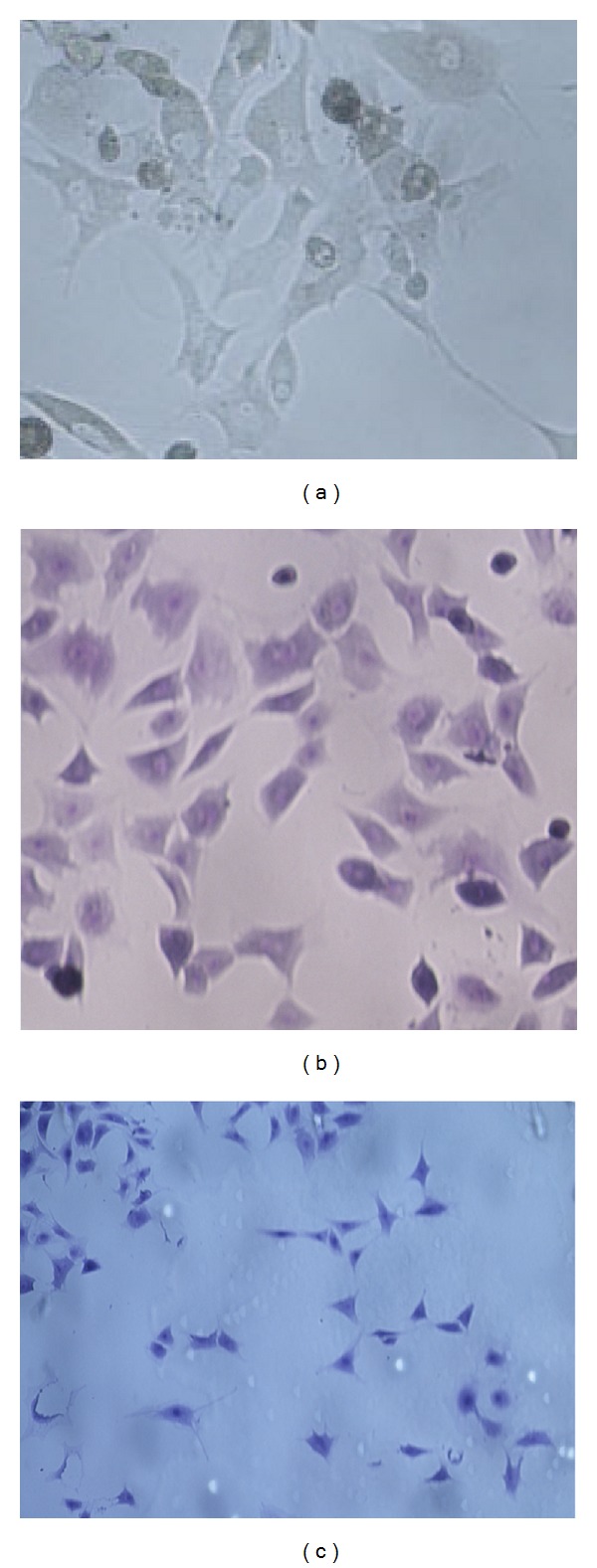
Identification of chondrocytes. Chondrocytes were stained with toluene blue (b) and type II collagen immunohistochemistry reaction (c). (a) was the unstained group cells derived from OA model.

Chondrocytes of passage 1 were inoculated onto 96-well plates. Three days later, toluene-blue staining revealed the synthesis of chondroitin sulfate, and immunohistochemical staining for type II collagen revealed that cells exhibited dark-brown cytoplasm, indicating that cells express type II collagen with no dedifferentiation. Both procedures gave positive staining in the separation cells, demonstrating the identification of chondrocytes (Figure 1).

**Figure 2:**
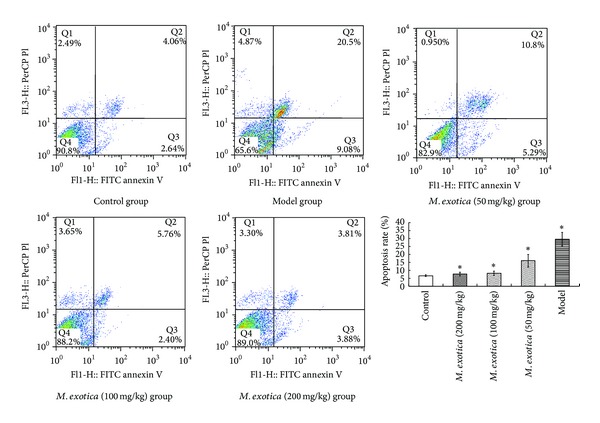
Inhibition of apoptosis by *M. exotica*. The OA chondrocytes were incubated in media containing different doses of *M. exotica* for 24 h. Model group was the OA chondrocytes incubated in normal media without adding any medicines. Control group was the healthy chondrocytes separated from normal rats and incubated in normal media. Cells were collected, and the amount of apoptotic cells was determined by flow cytometry using FITC annexin V/PI staining. The right histogram was the summarized data indicating the rate of apoptotic cells, as detected by flow cytometry. Data were presented by mean ± standard deviation of 4 replicates. *P < 0.05 as compared with control.

Annexin V is a type of Ca^2+^-dependent phospholipids-binding protein that can specially bind with high affinity to the phosphatidylserine of the cell membrane after it has been reversed during the process of apoptosis. Propidium iodide (PI) is a nucleic acid dye that cannot normally pass through the intact cell membrane, but in the middle and late stages of apoptosis, it can stain the nucleus due to breaks in the cell membrane. Flow cytometry using the FITC annexin V/PI double-staining method was used to generate an apoptotic cell scatter plot of different doses of *M. exotica* groups (Figure 2). Chondrocytes apoptosis could be significantly inhibited by *M. exotica*. At the doses of 100 mg/kg and 200 mg/kg of *M. exotica*, the situations were almost as moderate as that in the control group. In contrast, the model group showed the apoptosis rate as 29.55% (Figure 2).

### 3.3. Effects of *M. exotica* on Viability

**Figure 3:**
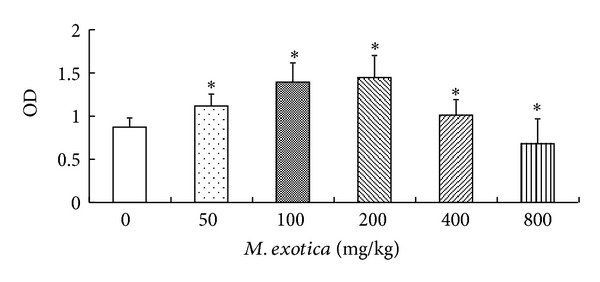
The effects of *M. exotica* on chondrocytes viability as determined by the MTT assay. Data were presented by mean ± standard deviation of 4 replicates. *P < 0.05 as compared with control.

The chondrocytes toxicities at 800, 400, 200, 100, 50, and 0 mg/kg of *M. exotica* were assessed by MTT assay. *M. exotica* with concentrations higher than 400 mg/kg had toxic effects on chondrocytes (Figure 3). However, the viability of the chondrocytes incubated with 400 mg/kg *M. exotica* was much less than that with 200 mg/kg *M. exotica*. As a result, 200, 100, and 50 mg/kg *M. exotica* were selected as the high, medium, and low concentrations, respectively; the criterion was used in subsequent experiments.

### 3.4. Changes in Expression of **β*-Catenin* and *COX-2 *Genes and the Apoptotic gene *Caspase 3* after Treatment with *M. exotica*

**Figure 4:**
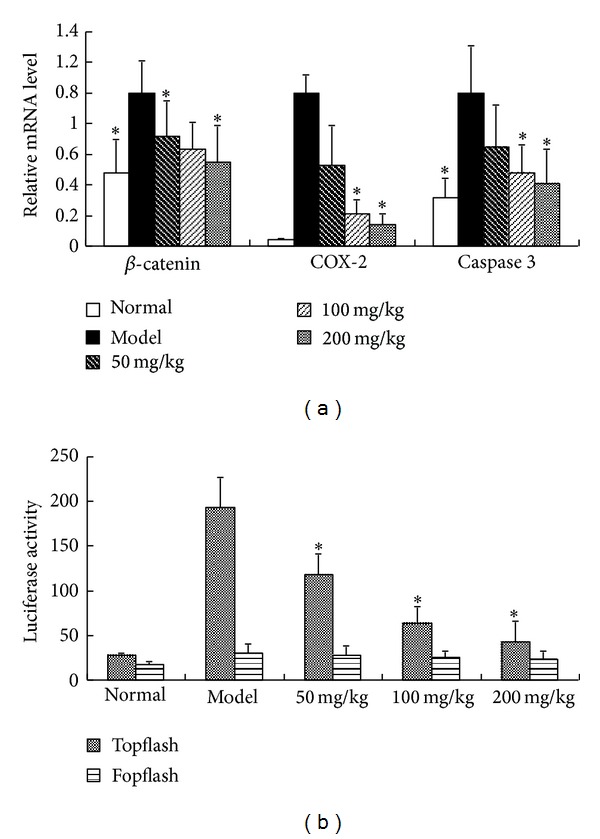
(a) Changes in **β*-catenin*, *COX-2*, and *caspase-3* mRNA expression in the control and model groups and in the groups treated for 24 h with 50 mg/kg, 100 mg/kg, and 200 mg/kg *M. exotica*. qRT-PCR was used to detect changes in mRNA expression of these genes. GAPDH was used as internal control. These data were representative of results obtained from the analysis of three independent experiments. Data were presented by mean ± standard deviation of 4 replicates. *P < 0.05 as compared with model. (b) Chondrocytes were transfected with Fopflash or Topflash luciferase reporters. Transfected cultures were treated with 50 mg/kg, 100 mg/kg, and 200 mg/kg *M. exotica* for 24 h. Data were ratios of firefly luciferase units from the respective reporters to constitutive CMV-regulated Renilla luciferase units normalized to their respective model group cultures. Data were presented by mean ± standard deviation of 4 replicates. *P < 0.05 as compared with control.

The Wnt/*β*-catenin signaling pathway had been reported to be associated with chondrocyte apoptosis [10]. To determine the possible pathways leading to apoptotic inhibition by* M. exotica*, the mRNA expressions of *β*-catenin signaling-associated genes **β*-catenin*, *COX-2*, and the apoptotic effecter gene *caspase 3* were assessed using qRT-PCR (Figure 4(a)). The **β*-catenin*, *COX-2*, and *caspase-3* mRNA levels of chondrocytes exposed to 200 mg/kg *M. exotica* were significantly different from those of the control group. The expression of *COX-2*, a target gene of *β*-catenin signaling, did decrease with exposure to increasing doses of *M. exotica* in a dose-dependent manner (Figure 4(a)). In cultures transfected with Fopflash or Topflash reporters, treatment with 50 mg/kg, 100 mg/kg, and 200 mg/kg of *M. exotica* for 24 h caused a significant decrease in Topflash activity compared to Fopflash (encodes mutated LEF/TCF binding sites) activity, indicating that *M. exotica* elicited a significant decrease in *β*-catenin regulated-reporter activity in chondrocyte (Figure 4(b)). Collectively, these results suggest that *M. exotica* alters chondrocytes *caspase-3* mRNA levels, possibly due to a *β*-catenin-dependent mechanism.

### 3.5. Change in Protein Expression of *β*-Catenin and COX-2 and the Apoptotic Effecter Caspase 3 in Chondrocytes

**Figure 5:**
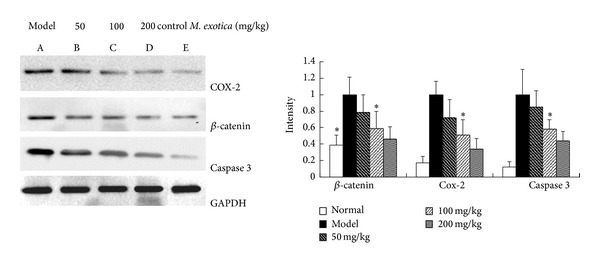
Changes in protein expression of *β*-catenin, COX-2, and caspase 3 in the model and control groups and in the groups treated for 24 h with 50 mg/kg, 100 mg/kg, and 200 mg/kg *M. exotica*. Western blot was used to determine changes in protein expression. These data were representative of results obtained from the analysis of three independent experiments. Data were presented by mean ± standard deviation of 4 replicates. *P < 0.05 as compared with model.

After chondrocytes were treated with *M. exotica* (50 mg/kg, 100 mg/kg, and 200 mg/kg) for 72 h, Western blot analysis was used to measure the expression of the *β*-catenin signaling-associated proteins *β*-catenin and COX-2 and the apoptotic effecter protein caspase 3. The protein expressions of *β*-catenin and COX-2 and the apoptotic effecter caspase 3 in condrocytes were dose-dependently downregulated by exposure to *M. exotica*, compared to the model group (Figure 5).

## 4. Discussion

*M. exotica*, a variety of* M. paniculata*, is known as an ornamental and hedge plant for its pleasant smell and beauty. It was upgraded to be a species, paralleled with the later, by a Chinese botanist in 1978. Both *M. exotica *and* M. paniculata* can be apparently distinguished from *M. koenigii* by the presence of yuehchukene and the absence of girinimbine in the roots [11]. Phytochemical studies reveal that coumarins and flavanoids are the two kinds of main components in the leaves of *M. exotica*. The coumarins include murrangatin, meranzin, phebalosin, isomurralonginol, umbelliferone, and scopoletin [12]; the flavanoids include 3,3′,4′,5,5′,6,7-heptamethoxyflavone, bannamurpanisin, exoticin, gardenin A, gardenin C, and gardenin E [13].

OA may be of unknown origin (idiopathic, primary) or related to a known medical condition or event. There is now strong evidence that the structural changes globally observed in OA are due to a combination of factors, ranging from mechanical to biochemical [14]. It is increasingly apparent that chondrocytes have the capacity to produce a variety of cytokines and mediators associated with inflammation [15]. These molecules influence a wide range of biological processes that include proliferation, differentiation, migration, and apoptosis.

TNF*α* and IL-1 are proinflammatory cytokines, which are associated with cartilage degeneration, synovial inflammation, and bone changes. IL-1*β* is known as playing a pivotal role to trigger apoptosis, which leads to further cartilage degradation. Chondrocytes stimulated with IL-1*β* *in vitro* have been used to mimic the microenvironment that occurs in OA [16]. Measuring a wide panel of mediators in the SF of both control and end-stage OA, Beekhuizen confirmed the involvement of inflammatory processes in OA [17]. IL-1*β* stimulus enhances the expression of paracrine pro-inflammation, including TNF*α* and IL-1*β* genes expression, which provides evidence for a positive feedback loop [18]. *M. exotica* has been reported to exhibit chondroprotective activity by decreasing the contents of TNF*α* and IL-1*β* in rat serum [7]. On one hand, PGE_2_ can upregulate NF-*κ*B through EP4/G protein/MAPK signaling to promote pro-inflammatory factors expressions; on the other hand, PGE_2_ is the product of COX-2, a target gene of Wnt/*β*-catenin pathway, which is the classical target of NSAIDs available for OA treatment. To study on *M. exotica* modulating Wnt/*β*-catenin pathway in OA chondrocytes, indomethacin was identified as the positive control. In this study, pro-inflammatory cytokines in OA SF were further investigated, and *M. exotica* was demonstrated to exhibit downregulation of TNF*α* and IL-1*β* expression, although not statistical significant difference. *In vitro*, the separated OA chondrocytes were cultured with rat serum containing different doses of *M. exotica*, and then the chondrocytes apoptosis with FITC annexin V/PI double staining were evaluated by flow cytometry. It demonstrated that *M. exotica* could significantly protect chondrocytes from initiating apoptotic processes in a dose-dependent manner. To support this result, MTT assay was employed. The viability of chondrocytes, incubated with different concentrations of *M. exotica*, was showed in dose-dependent manner, which was consistent with that by flow cytometry.

It is shown that both constitutive up- or downregulation of the canonical Wnt pathway negatively influence cartilage development and maintenance resulting in OA-like features [19]. This suggests that a tight regulation of this signaling cascade is crucial throughout the chondrocyte life cycle. *β*-catenin is a key molecule in the canonical Wnt signaling pathway and plays a critical role in multiple steps during chondrocyte formation and maturation. Several drugs and synthetic or natural compounds have been reported to inhibit and/or modulate *β*-catenin signaling [20]. However, their detailed mechanisms are little understood. These small-molecule inhibitors may act by reducing *β*-catenin stability [21], blocking *β*-catenin-TCF interaction [22] or *β*-catenin-CREB binding protein interaction [23], stabilizing Axin2 level [24], preventing dishevelled-Frizzled interaction [25], or other indirect inhibition [20]. For instance, inhibitor of *β*-catenin and T-cell factor (ICAT) is an 82-amino-acid small molecule [26] whose crystal structure reveals binding capacity to the armadillo repeats of *β*-catenin. This binding disrupts the complex formation of *β*-catenin with TCF/LEF [26, 27] and thus leads to inhibition of signaling in this pathway. FRZB encodes sFRP-3, a glycoprotein that antagonizes the signaling of Wnt ligands through Frizzled membrane-bound receptors. *In vitro* transfection assays demonstrated that sFRP-3 could inhibit *β*-catenin nuclear translocation and TCF/LEF-dependent transcriptional activation [28]. Rodriguez et al. proved that *COX-2* gene expression was transcriptionally modulated by the *β*-catenin-TCF/LEF pathway, and *β*-catenin was bound to AU-rich elements (ARE) in the 3′-UTR of COX-2 mRNA and stabilized the mRNA [29]. Quercetin is demonstrated to antagonize the Wnt signaling pathway via disrupting the association of *β*-catenin with TCF/LEF-1 [30]. The main constituents in *M. exotica* are flavones and coumarins, and most of flavones are quercetin analogues. We found that *M. exotica* significantly downregulated mRNA and protein expressions of *β*-catenin and COX-2 and reporter activity. However, the detail mechanism is yet to be investigated.

COXs catalyze the conversion of arachidonic acid to prostaglandin H2 (PGH_2_), which is then further processed to PGE_2_, PGI_2_, PGD_2_, or thromboxane A_2_ by specific synthases. In general, increased COX-2 levels are associated with augmented PGE_2_ production. Goessling reported that PGE_2_ modified the wnt signaling cascades at the level of *β*-catenin degradation through cAMP/PKA-mediated stabilizing phosphorylation events [31]. Previous study shows that *M. exotica* exhibits significant chondroprotective activity by decreasing the expressions of iNOS, IL-1*β*, and TNF-*α* *in vivo*, and antinociceptive activity in animal models of acetic acid induced writhing response, hot-plate latent pain response test, carrageenan-induced hind paw edema, and xylene-induced ear edema [7]. However, there is no direct evidence exists for PGE_2_ positive feedback to *β*-catenin signaling by *M. exotica* in chondrocytes.

Conclusively, *M. exotica* decreased the contents of TNF*α* and IL-1*β* in rat OA SF and the chondrocytes apoptosis *in vitro*, probably due to inhibiting *β*-catenin signaling.

## Figures and Tables

**Table 1:** Primer sequences for different genes.

β-catenin	Forward	5′-ACAGCACCTTCAGCACTC T- 3′	[32]
	Reverse	5′-AAGTTCTTGGCTATTACGACA- 3′	

COX-2	Forward	5′-GCACAAATATGATGTTCGCATTC- 3′	[33]
	Reverse	5′-CAGGTCCTCGCTTCTGATCTG- 3′	

caspase 3	Forward	5′-CTCGGTCTGGTACAGATGTCGATG- 3′	[34]
	Reverse	5′ -GGTTAACCCGGGTAAGAATGTGCA- 3′	

GAPDH	Forward	5′-CAGTGGCAAAGTGGAGATTG- 3′	[9]
	Reverse	5′-AATTTGCCG-TGAGTGGAGTC- 3′	

**Table 2:** Synovial fluid lavage biomarkers (dilution adjusted by comparing the urea concentration in SF). All values provided as the mean ± standard deviation (*n* = 10).

Biomarkers	Control group	Model group	Indomethacin group	*M. exotica* group		
				50 mg/kg	100 mg/kg	200 mg/kg
IL-1*β* (pg/mL)	36.4 ± 12.1	89.2 ± 14.8	62.2 ± 8.6	68.5 ± 10.3	53.3 ± 10.8	48.3 ± 9.4
TNF-*α* (pg/mL)	33.6 ± 9.5	80.3 ± 11.6	57.4 ± 11.8	61.3 ± 15.2	50.5 ± 11.4	47.2 ± 12.5
